# Exploration Editors’ Favorite Research in 2025

**DOI:** 10.1002/exp2.70143

**Published:** 2026-03-05

Exploration editors select their favorite research articles from 2025, reflecting advances and impactful contributions across diverse research areas.

## 1 ^195^Pt NMR for Pt Single‐Atom Catalyst Characterization

Resolving local metal coordination with molecular precision is challenging for single‐atom catalysts (SACs), as conventional techniques yield only average structural information. A recent study combined ^195^Pt solid‐state NMR spectroscopy with Monte Carlo simulations to precisely characterize the coordination environments of Pt SACs [1]. By acquiring ultra‐wideline NMR spectra and converting them into quantitative “SAC signatures” via simulations, the approach enabled tracking of site evolution during synthesis and catalysis, providing guidance for reproducible SACs development. It also points to extension beyond SACs to dual‐atom/single‐cluster architectures and other NMR‐active metals.

## 2 Precision Nucleic Acid Targeting

Sequence‐specific recognition of double‐stranded nucleic acids has long been constrained by CRISPR–Cas systems’ PAM motif dependence and off‐target effects, limiting applications in diagnostics and imaging. A study addressed this gap with the λExo–pDNA system: bacteriophage λ exonuclease and a 5′‐phosphorylated DNA guide enable PAM‐independent targeting of dsDNA and DNA–RNA duplexes [2]. The system exhibited high mismatch sensitivity and Mg^2+^‐dependent pDNA digestion, supporting pathogen detection, mutation screening, and genomic imaging without motif restrictions.

## 3 Enzymatic Recycling of Thermoset Polyurethanes

Enzymatic recycling strategies for thermoset polyurethanes offer a solution to the environmental burden of plastic waste, avoiding the energy‐intensive conditions of chemical recycling. Chen et al. introduced GRASE, a GNN‐based framework, to discover glycolysis‐compatible urethanases like AbPURase—exhibiting hundreds‐fold higher activity in harsh solvents [3]. This breakthrough enables kilogram‐scale, near‐complete depolymerization, driving industrial adoption of eco‐friendly polyurethane recycling.

## 4 Coherent Quantum Communications in Deployed Telecom Networks

Coherent quantum communication protocols have long been limited by specialized hardware like cryogenic detectors and ultra‐stable cavities, hindering integration with existing telecom infrastructure. A recent study implemented twin‐field quantum key distribution (TF‐QKD) over a 254 km commercial network, using off‐band phase stabilization and non‐cryogenic APD detectors [4]. Achieving 110 bits per second key distribution, the study doubled the practical QKD distance and realized a measurement‐device‐independent quantum network.

## 5 Real‐Time In Situ Magnetization Reprogramming for Soft Robotics

Magnetic soft robots have long been constrained by the inability to reprogram magnetization profiles on demand, limiting their versatility. A recent bioinspired approach enabled real‐time in situ magnetization reprogramming, rearranging internal magnetic units to produce varied magnetization profiles [5]. This bioinspired work rearranged internal magnetic units to generate varied magnetization profiles, supporting 1D‐to‐3D deformations and validating applications in contact‐free navigation, reprogrammable cilia arrays, coordinated multi‐instrument operation, and shape‐adaptable gripping.

## 6 Computational Design of Superstable Proteins

Generating proteins with nanonewton‐level mechanical stability and extreme thermal resistance is a major challenge in the field. AI‐guided structure/sequence design was recently integrated with all‐atom molecular dynamics simulations to engineer superstable proteins via maximized hydrogen bonding [6]. The computational work expanded force‐bearing β‐strands, increasing backbone hydrogen bonds from 4 to 33, and validated candidates with unfolding force tests and thermal stability assays, yielding proteins like F553 with unfolding forces exceeding 1000 pN.

## 7 NeuroWorm: Movable Long‐Term Implantable Soft Microfibre for Dynamic Bioelectronics

Implantable bioelectronics have traditionally been confined to fixed positions, limiting dynamic targeting and long‐term biocompatibility. A standout 2025r study introduces NeuroWorm, an earthworm‐inspired soft and stretchable microfibre, tackling the core challenge of movable, minimally invasive long‐term implantation through magnetic control and a rolled 2D‐to‐1D design [7]. This innovation opens avenues for neural monitoring, neuromodulation, and human‐machine interfaces with stable signal acquisition and enhanced biocompatibility.

## 8 Oral Delivery of Liquid mRNA Therapeutics

The development of oral mRNA therapeutics has long been hindered by the belief that gastric degradation would inactivate mRNA before it reached the intestines. Addressing this critical challenge, a recent study presents RNACap, an engineered capsule designed for oral delivery of liquid mRNA therapeutics, addressing the key challenge of protecting fragile mRNA from the harsh gastrointestinal (GI) tract [8]. This study opens the door to a future where mRNA therapies—whether for diseases or vaccines—could be as simple as swallowing a pill.

## 9 Organic Ionic Plastic Crystals for Sustainable Refrigeration

Current barocaloric materials face issues of inappropriate transition temperatures and high driving pressures. Recent work introduced organic ionic plastic crystals as a new family of barocaloric materials, with subambient solid–solid transition temperatures, colossal entropy changes (92 to 240 J kg^−1^ K^−1^), and high‐pressure sensitivity [9]. Tunable via ion structural modification, they achieve low reversible pressures and substantial refrigeration capacities, promising energy‐efficient, sustainable cooling alternatives.

## 10 Single‐Cell Chromatin Accessibility Profiling of Early Embryos

Studying chromatin accessibility during mammalian preimplantation development has long been limited by short‐read sequencing and scarce cell availability. The scNanoATAC‐seq2 method, a long‐read single‐cell assay, overcomes these challenges, mapping the chromatin landscape of mouse embryos from zygote to blastocyst [10]. The approach revealed lineage‐specific transcription factor dynamics, allele‐specific X‐chromosome inactivation, and cis‐regulatory roles of repetitive elements, resolving individual copies missed by conventional methods.
